# Studies of adsorption of α,β-unsaturated carbonyl compounds on heterogeneous Au/CeO_2_, Au/TiO_2_ and Au/SiO_2_ catalysts during reduction by hydrogen†

**DOI:** 10.1039/d1ra09434c

**Published:** 2022-02-15

**Authors:** Maciej Zielinski, Wojciech Juszczyk, Zbigniew Kaszkur

**Affiliations:** Institute of Physical Chemistry, Polish Academy of Sciences ul. M. Kasprzaka 44/52 01-224 Warszawa Poland mzielinski@ichf.edu.pl; National Centre for Nuclear Research, NOMATEN Centre of Excellence ul. A. Soltana 7 05-400 Otwock-Swierk Poland

## Abstract

Our research focuses on phenomena accompanying adsorption of mesityl oxide (4-methylpent-3-en-2-one) on the surface of heterogeneous supported gold catalysts: Au/CeO_2_, Au/TiO_2_ and Au/SiO_2_. We have studied reduction in the gas phase of (volatile) α,β-unsaturated carbonyl compounds (R-(V)ABUCC) which mesityl oxide is a basic model of. *In situ* infrared (IR) spectroscopy was employed to establish that the most active catalysts allow adsorption of conjugated ketones or aldehydes in the enolate (*i.e.* bridge-like adsorption through the oxygen from the carbonyl group and the β-carbon) and carboxylic form or with the ^α^C=^β^C double bond on a Lewis acidic site. Reductive properties of the catalysts and pure supports were studied by temperature-programmed reduction (TPR). We show that cerium(iv) oxide (CeO_2_, ceria) and titanium(iv) oxide (TiO_2_, titania) when decorated with gold nanoparticles (AuNP) can interact with hydrogen at temperatures approx. 150 °C lower than typical for pure oxides what includes even cyclic adsorption and instant release of H_2_ below 100 °C in the case of gold–ceria system. Morphology and structure characterisation by transmission electron microscopy (TEM) and powder X-ray diffraction (PXRD) confirms that, with the obtained Au loadings, we achieved excellent dispersion of AuNPs while maintaining their small size, preferably below 5 nm, even though the Au/CeO_2_ catalyst contained broad distribution of AuNPs sizes.

## Abstract 2

We deliver spectroscopic IR data describing the adsorption phenomena accompanying reduction of conjugated carbonyl compounds aided by heterogeneous catalysts.

## Graphical abstract

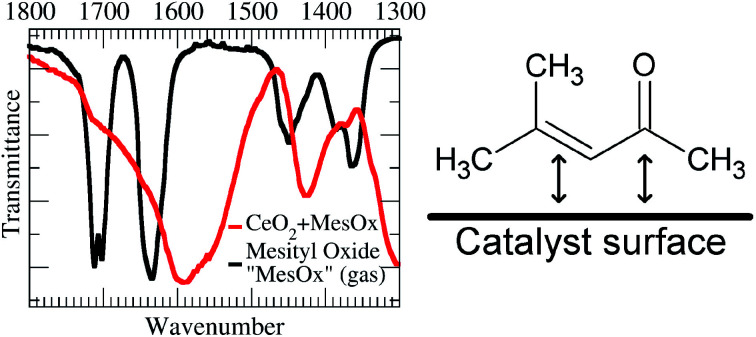

## Introduction

The development of the technology of the next generation of catalysts, especially the promising gold (Au) catalysts, and their prospective application in industrial processes depend now on the knowledge explaining why the desired properties occur. This research focuses on heterogeneous nanocrystalline Au (AuNCr) catalysts based on oxide supports: silica (SiO_2_), ceria (cerium(iv) oxide, CeO_2_) and titanium(iv) oxide (TiO_2_). These systems exhibit different chemical properties in reduction of (volatile) α,β-unsaturated carbonyl compounds (R-(V)ABUCC) with gaseous hydrogen. We believe that mutual influence of support on gold and *vice versa* provides occurrence of desired properties (such phenomenon is referred to as metal–support interaction, MSI^1–3^). Utilisation of gold-based systems (*e.g.* ref. 4) creates additional solution, with regard to existing ones (*e.g.* ref. 5–10), to precisely control the hydrogenation pathway and, thus, to obtain desired products. The main scope of this work is to describe surface adsorption phenomena by *in situ* infrared (IR) spectroscopy that can later be utilised for studies of catalyst surface structure changes by means of various structure-sensitive techniques arranged as *in operando* setups, including *in operando* electron microscopy,^11^*in operando* powder X-ray diffraction^12,13^ and others *e.g.* ref. 14.

**Fig. 1:**
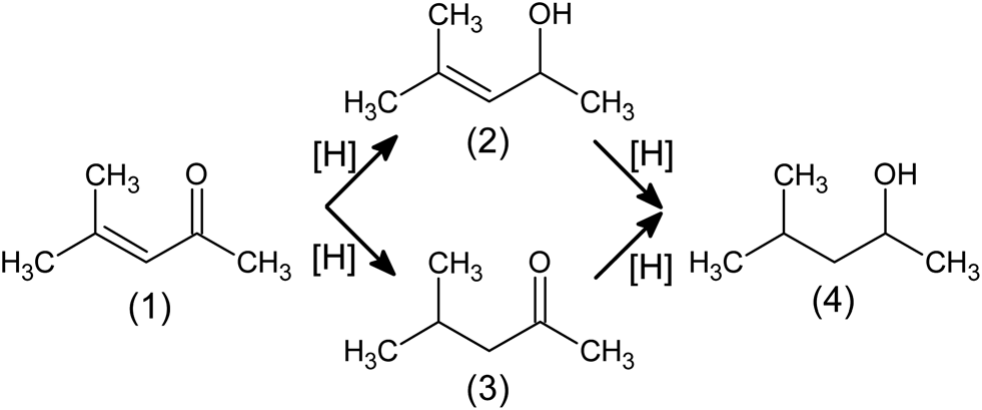
Hydrogenation pathways of mesityl oxide (4-methylpent-3-en-2-one, (1)) to its derivatives: 4-methylpent-3-en-2-ol (2); 4-methylpentan-2-one (3); 4 methylpentan-2-ol (4).

Hydrogenation of conjugated unsaturated carbonyl compounds (*i.e.* aldehydes and ketones) is the process of great importance in which valuable commodities are obtained, to mention just printer inks,^15^ perfumes^5,16^ and medicines.^17^ In the first step (Fig. 1), unsaturated alcohol (so called allylic alcohol) or saturated carbonyl compound are produced. Next step leads always to the same product: saturated alcohol. Allylic alcohols production is of special interest of manufacturers, as currently these compounds are synthesised in costly stoichiometric reactions with NaBH_4_, Grignard compounds or homogeneous catalysts containing heavy metals that can assure *e.g.* high enantiomeric purity.^17^ Although gold catalysts exhibit desired properties, exhaustive answers covering ensuring long-time stability of catalysts and key physicochemical features responsible for observed phenomena are still not fully provided.

Reaction in the gas phase, as chosen for this project, has numerous advantages. Lack of solvent eliminates the necessity of taking it into account in preparation of the *in situ* experiment and during data analysis what applies especially to issue of useful analytical signal shielding or introduction of additional background. It is also beneficial from the point of view of future technology: the process as a whole potentially can be designed to be more economically efficient. It is also more feasible to scale it up to the required production yield. The gaseous stream of products is ready as such to be fractionated by temperature-controlled condensation into subsequent collectors. The catalyst itself is less prone to mechanical degradation what increases its durability necessary during time-consuming laboratory experiments or factory plant operation. Selected systems containing different supports allow tracking different surface phenomena.

There have been already described some mechanisms addressing observed gold activity, *e.g.* ref. 18 and 19. The already mentioned strong dependence of gold catalytic activity on the used support suggests Mars–van Krevelen reaction mechanism what was confirmed in the literature.^20–22^ In a two-molecule reaction, one of the molecules adsorbs on the metal nanocrystal and the other on the surface of the support, but close to the interface between them. In such case, the support plays a significant role in the reaction mechanism and, thus, in selectivity and overall performance of the catalyst.

*In situ* IR spectroscopy gives insight into adsorption phenomena happening on the catalyst surface. Differential IR spectrum of the pure catalyst and exposed to vapours of volatile unsaturated carbonyl compound (we used mesityl oxide, 4-methylpent-3-en-2-one, Fig. 1, as a model molecule) shows how this organic compound is adsorbed on the catalyst surface and how it behaves in the presence of hydrogen. If chemical adsorption occurs, the spectrum will be significantly changed with respect to the spectrum for gaseous mesityl oxide and new bands will be visible.

IR spectroscopy investigation was backed-up with structural characterisation of the catalysts by electron microscopy and powder X-ray diffraction to validate the syntheses results. Additionally, the behaviour of the materials under purely reductive conditions was examined using temperature-programmed reduction.

Development of efficient and applicable catalytic materials shall focus on creating adsorption sites on the materials' surface that favour hydrogenation of just right functional groups to desired final products. Herewith, we provide a useful recipe how to study adsorbates on powder materials. Selected conditions can be screened readily, like temperature, reagents concentration or pressures. Equally important, we supply the method to process data and draw conclusions. Investigation of a series of materials should finally end up in finding the optimised system working under appropriate conditions (to be established separately).

An important issue, reminded in Conclusions section, is the possible occurrence of limiting reaction steps, *e.g.* desorption of final products. Such an obstacle may cause a very promising catalyst, although able to provide right circumstances for the reagents to be transformed, to fail in supplying large quantities of the desired products at the reactor's outlet, thus, allowing the less favourable product to be obtained instead.

On balance, the results of this research show effects of interaction in which reagents, intermediate products and the catalysts surface (both the surface of the support and crystals of gold) are involved. Insights and new data on the studied catalytic systems are likely to be the crucial step towards development of this branch of chemistry and steer further research activities.

## Experimental

### Materials

Mesityl oxide (abbrev. MesOx, 99.5%, Merck KGaA) for catalytic activity tests was stored in the Schlenk flask under He. MesOx for *in situ* FTIR spectroscopy experiments was additionally distilled under N_2_ atmosphere and was stored in the Schlenk flask under only its own vapours (*i.e.* at a diminished pressure).

The following gases: H_2_ (99.999%, Multax S.C.), He (99.999%, Multax S.C.), N_2_ (99.999%, Multax S.C.), Ar (99.999%, Multax S.C.), 10%_vol_ H_2_ in Ar (prepared by Multax S.C. from 99.999% purity gases), O_2_ (99.999%, Multax S.C.) were used in all experiments.

In the catalysts syntheses the following materials were used: cerium dioxide (CeO_2_, Austranal Praeparate), titanium dioxide (TiO_2_, Aeroxide® P25), silica (SiO_2_, Davison 62) hydrogen tetrachloroaurate(iii) trihydrate (HAuCl_4_·3H_2_O, 99.9%, Alfa Aesar), sodium borohydride (NaBH_4_, 99%, Sigma-Aldrich), sodium hydroxide (NaOH, ppa, Chempur), 1,2-ethylenediamine (H_2_NCH_2_CH_2_NH_2_, will be abbreviated by “en”, ≥99.5%, Roth), diethyl ether ((CH_3_CH_2_)_2_O, will be abbreviated by “Et_2_O”, ppa, Chempur), propan-2-ol (ppa, Stanlab), 25% ammonia solution (NH_3 aq_, ppa, POCH) and double-distilled water (also referred to as redistilled water).

### Catalysts syntheses

8.7%_wt_ Au/CeO_2_ (composition assessed by XRF measurements) catalyst synthesis was inspired by M. Haruta recipe^23^ and M. Zielinski MSc Thesis^24^ modified by the introduction of the final reduction step with NaBH_4_.

20 ml 0.0254 M (0.82%_wt_) of tetrachloroauric acid solution diluted with 330 ml of redistilled water was vigorously stirred at 500 rpm in darkness. Under constantly monitored pH value which never exceed pH = 11, it was alkalized dropwise with 51 ml of 0.05 M NaOH_aq_ and was left overnight under stirring. The initially yellow solution turned into transparent. This indicated the tetrahydroxyaurate (Au(OH)^4−^) complex was formed successfully. Then, 900 mg of CeO_2_ which was suspended in 50 ml of redistilled water *via* ultrasonication aided by mechanical stirring was added to the tetrahydroxyaurate complex solution. The mixture was further stirred for 2 h at 80–85 °C. After cooling the mixture down to 0 °C, 58 mg of NaBH_4_, dissolved in 20 ml of 0.1 M NaOH_aq_, was added dropwise. It resulted in immediate colour change into purple. The mixture was subsequently stirred for another 12 h in room temperature. Then, the catalyst was filtered and rinsed 15 times with redistilled water until no chlorine anions were detected. It was finally dried overnight under vacuum in 40 °C. A ready-for-use catalyst was stored in darkness in a desiccator.

0.5%_wt_ Au/TiO_2_ catalyst synthesis was also inspired by M. Haruta recipe^23^ and followed the method described in M. Zielinski MSc Thesis^24^ which did not include the final reduction step with NaBH_4_, but assumed spontaneous reduction of gold cations by contact with air during drying of the catalyst at diminished air pressure.

To obtain approx. 1800 mg of the material, 40 ml 0.0254 M (0.82%_wt_) of HAuCl_4_ solution diluted with 337 ml of redistilled water was vigorously stirred at 600 rpm in darkness. While the pH value was on-line monitored and never exceeded 11, gold complex with hydroxyl groups was formed by alkalisation with 100 ml of NaOH_aq_ added dropwise followed by stirring of the whole solution overnight. The solution turned from yellow into transparent. Later, it was poured into suspension containing 1800 mg of TiO_2_ in 100 ml of redistilled water stirred at 500 rpm. The resulting constantly stirred mixture was heated to 80–85 °C and maintained in this temperature range for 3 h. The suspended TiO_2_ powder having immobilised on its surface the gold hydroxy-complex was filtered and rinsed 10 times with redistilled H_2_O, while maintaining 50 °C, until no chlorine anions were detected. It was finally dried for 48 h under diminished air pressure at 40 °C. A ready-for-use catalyst was stored in darkness in a desiccator.

10.0%_wt_ Au/SiO_2_ catalyst synthesis recipe was based on the idea of Y. L. Lam and M. Boudart^25^ which was applied so that only gold containing catalyst was obtained (originally Au–Pd alloyed catalyst's active phase was obtained). Gold precursor was prepared according to original B. P. Block and J. C. Bailar^26^ recipe which was re-examined by W. J. Louw and W. Robb^27^ and optimised by K. Kitada.^28^

Solutions of 1 g of hydrogen tetrachloroaurate(iii) trihydrate in 10 ml of diethyl ether and 1 ml of 1,2-ethylenediamine in 5 ml of Et_2_O were mixed. A gummy yellow precipitate was formed. Following 30 min of refluxing, the solvent was evaporated under diminished pressure. An orange solution of the product was obtained after addition of approx. 10 ml of water. Following addition of approx. 90 ml of propan-2-ol a yellowish precipitate was formed and subsequently filtered. Crystallization of di(ethylenediamine)gold chloride from water and propan-2-ol mixture was repeated resulting in white powder which was dried overnight under vacuum at 40 °C. Recrystallization was applied every time before using the gold complex in catalyst's synthesis.

All of the following steps were performed in darkness. In order to prepare 1000 mg of the catalyst, 900 mg of SiO_2_ was suspended in 0.4 ml of 25% ammonia solution diluted with 50 ml of water resulting in pH ∼ 11. The mixture was stirred at 500 rpm. 215 mg of fresh di(ethylenediamine)gold chloride dissolved in 50 ml of water (transparent solution) was added dropwise to silica suspension. The slurry was heated up to 70 °C. Following 1 h when the temperature never dropped below 68 °C, the mixture, which turned into yellow, was filtered under diminished pressure, rinsed 10 times with water and dried overnight under vacuum. A yellow powder was obtained.

Before performing any experiments, the catalyst was activated in 20 ml min^−1^ flow of H_2_ for 80 min in 50 °C, then 160 min in 100 °C and finally 400 min in 150 °C. The activation procedure aimed at reducing the gold cation complex anchored to SiO_2_ surface to gold particles closely bound with the support surface. At the same time, the 1,2-ethylenediamine ligand was released as possibly free ammonia (NH_3_) and ethane (C_2_H_6_).

### The catalytic activity tests

The catalysts' chemical activity was checked in the reaction setup based on the U-shaped glass reactor resembling the model of the Plug-Flow Reactor (PFR). The catalyst's bed (approx. 100 mg depending on the material's bulk density) was formed as a thin layer of powder uniformly distributed over the 15 mm fused silica disk welded into the reactor's walls. The whole reactor was placed in the furnace that was slowly (2 °C min^−1^) heated from 145 °C up to 350 °C and all the tubing outside the furnace through which MesOx vapours flew were maintained at 145 °C (*i.e.* at lowest reliable temperature at which neither MesOx nor its hydrogenation derivatives condense).

16.93 μl min^−1^ of liquid MesOx (equivalent to 3.33 ml min^−1^ flux after evaporation to the gas phase) was dosed using a syringe pump (New Era Pump Systems 1000 X-ES) into the gas stream formed by mixing 4 ml min^−1^ of H_2_ with 14 ml min^−1^ of He at 1 atm. The 20% H_2_ excess was provided to allow detection of fully-hydrogenated products in case of full conversion of MesOx.

Right behind the glass reactor, the quartz capillary sampling the outlet stream to the mass spectrometer (MS, HPR-20 from Hiden Analytical Ltd.) was attached. The quadrupole MS with 2 detectors (Faraday cup and secondary electron multiplier covering together pressure range from 10^−13^ to 10^−5^ bar) provided on-line insight into the hydrogenation reaction outcome up to 200 amu. The unused reactant and all products were condensed and collected before the exhaust.

To avoid unwanted condensation/polymerisation reactions at highest temperatures and maintain stable long-time activity of the studied materials, the *in situ* IR experiments were carried out at *T* ≤ 300 °C (self-ignition temperature of *e.g.* MesOx in air is 344 °C (ref. 29) and of 4-methylpentan-2-ol is 305 °C (ref. 30)). The MS spectra analysis results were validated by gas chromatography (Agilent 8860, column ZB-WAX) analysis of the condensate collected only at 280 °C for 1 h (see ESI†).

### *In situ* infrared spectroscopy

Fourier-transformed infrared (FTIR) spectra in transmission mode were acquired using the Nicolet 6700 spectrometer equipped with a in-house made of quartz glass *in situ* measurement chamber with CaF_2_ windows.^24,63-65^ The chamber was connected to the tube furnace localised directly above the chamber. The specimen (thin pellet made of grinded and pressed powder) mounted in the quartz holder was lifted between the chamber for the spectrum acquisition and the furnace for adsorption/desorption of gases at a given temperature.

The setup was connected to a vacuum-gas system. Oil diffusion pump with a double liquid nitrogen trap provided vacuum approx. 2 × 10^−5^ mbar. H_2_ was stored in a glass tank and was dosed to the catalyst pellet in the furnace by adjusting the pressure to 50 mmHg in the *in situ* setup isolated from the vacuum system at that moment. Freshly distilled MesOx was dosed from the Schlenk flask attached directly to the glass apparatus.

Before the main part of *in situ* FTIR experiment, each sample (the catalyst or the pure oxide support) pellet was heated at 300 °C under vacuum for at least 1 h. The 300 °C temperature was maintained during later stages of the experiment. The background spectrum was the one collected for an empty chamber and it was subtracted from all subsequent spectra. The fresh preheated sample was lowered from the furnace to the chamber to acquire (after the sample cooled down) the spectrum corresponding to the clean catalyst's surface state “as it was”. Then, the MesOx vapours (partial pressure equalled saturation at room temperature) were supplied for 5 min to the previously vacuum-pumped system and the sample was placed at that time in the furnace set to 300 °C. Next, the system was vacuum-pumped for 15 min and the spectrum of MesOx adsorbed at the catalyst's surface was acquired. Later, 50 mmHg of H_2_ was supplied for 5 min to the sample with adsorbed MesOx, placed in the furnace. After that time, a spectrum corresponding to the absorbates state under H_2_ atmosphere was acquired. Finally, the system was vacuum-pumped again for 15 min and the spectrum of adsorbates remaining at the catalyst's surface was collected.

Thus, sets of 3 valuable spectra were obtained, corresponding to: (1) pure sample; (2) sample with adsorbed MesOx; (3) sample with adsorbed species after being in contact with H_2_. Data analysis was based on differential spectra, *i.e.* after spectrum no. 1 was subtracted from spectra no. 2 and 3 to reveal the contributions from the adsorbates.

### Temperature-programmed reduction

TPR measurements were carried out in a glass apparatus equipped with the U-shaped fixed bed quartz glass reactor, with the TCD detector and using 10%_vol._ H_2_ in Ar as the carrier gas. The catalysts and their pure supports underwent the same experimental procedure. At first, the samples were flushed with pure Ar for 1 h, after which the carrier gas was changed to H_2_/Ar mixture. Then, there were 2 repetitive cycles of temperature ramp (10 °C min^−1^) up to 250 °C (each heating cycle was followed by the cooling step down to room temperature under pure Ar). These cycles were intended to show whether low-temperature and instantly reversible interaction of the sample with gaseous H_2_ can be observed. Finally, the heating cycle up to 950 °C was performed to provide comprehensive characterisation of the materials' behaviour under reducing atmosphere.

In the case of Au/SiO_2_ catalyst, prior to carrying out the main TPR cycle, the fresh as-synthesised sample still containing Au–en complexes was subjected to the activation procedure *in situ* in the TPR apparatus without acquisition of the TCD signal. For the pure SiO_2_ sample, only one TPR cycle from room temperature to 950 °C was performed due to very inert character of SiO_2_ showed in the preliminary studies.

### Transmission electron microscopy

*Ex situ* Transmission Electron Microscopy (TEM) studies in the scanning mode (STEM) were conducted on TALOS F200X microscope with X-FEG gun operated at 200 keV and equipped with Super X EDS system used for elemental composition mapping.

## Results and discussion

### Catalytic activity

Each of the pure supports was expected to be inactive in R-(V)ABUCC reaction which indeed is the case. However, pure CeO_2_ shows, judging by the selected signals (refer to Fig. S1 in ESI†) in the MS spectrum acquired in function of the temperature (Fig. S2†) small and rapidly decreasing activity towards conversion of MesOx into a SK (saturated ketone, methyl-isobutyl ketone, 4-methylpentan-2-one). It is assumed, that such an observation is possible due to presence of residual adsorbed components of air that cannot be removed at 145 °C, which are the places of adsorption and the starting point for the reaction. It is doubtful that introduction of H_2_/He mixture to the reactor prior to starting dosing MesOx can explain the behaviour of CeO_2_, because no interaction between H_2_ and pure CeO_2_ was spotted at 145 °C in TPR studies (described later in the text).

Comparing the catalysts performance by GC analysis of R-(V)ABUCC reaction products collected over 1 h at 280 °C (Table S1†), 8.7%_wt_ Au/CeO_2_ proves to be the most active system. It converts almost 25% of α-MesOx from the stream mainly into SK (yield approx. 15%). 0.5% Au/TiO_2_ catalyst converts over 16% of α-MesOx, but mainly into its β isomer, and only about 3.5% of SK is formed. Similarly, 10.0%_wt_ Au/SiO_2_ converts only 9.7% of α-MesOx into, almost equal amounts of, SK (4.7%) and β isomer (5%).

Performance of the catalysts was not optimised because the chemical tests in the glass reactor were carried out to provide a reference point for the *in situ* FTIR studies (described below) and the experiments in the *in operando* PXRD chamber that are planned to be carried out as the next research step. Another, possibly important, factor was that a thin layer of material uniformly distributed over the fixed bed in the glass reactor had to be applied. This was crucial to maintain the differential glass reactor model and prevent any mass transport inside the sample layer, especially leading to a creation of concentration profile of gold particles AuNPs towards the direction of gas flow.

### *In situ* IR spectroscopy

The *in situ* collected FTIR spectra show that both: the support type and lack or presence of AuNPs are crucial factors determining which adsorbed forms of MesOx can be found on the surface of the studied material. After H_2_ is introduced to the system, some of the observed bands have their peak-tops shifted by a couple of wavenumbers (cm^−1^) what may be a trace of some intermediate products of hydrogenation of MesOx. A summary of the results described below and comparison with the literature can be found in Table 1.

**Table 1:** Comparison of the vibrational bands positions of functional groups found in mesityl oxide and acetone adsorbed on different materials. In parentheses “(+/−#)” the shift of a band after introduction of H_2_ (if observed) was given

Material	Vibrational band positions [cm^−1^]							Reference
	*ν*(C=O)		*ν*(C=C)		*ν*(–C(O)O–)	*ν*(O=C=C–)	*ν*(R_2_C=C–O^−1^)	
	Broensted site	Lewis site	Broensted site	Lewis site				
Mesityl oxide								
Au/SiO_2_	1732 (−14)	1677 (−9)		1609 (−2)				This work
SiO_2_	1735 (−1)	1678–1668 (−5)		1611–1607 (−1)				“
Au/TiO_2_	1708	1654			1539 (−1)		1588	“
TiO_2_	1701	1642			1550			“
CeO_2_	1714				1589–1500		1589 (+1)	“
HZSM-5	1617		1557					37 and 60
USY	1632–1631	1676	1565	1604				“
Al_2_O_3_		1673		1603–1589				“
TiO_2_					1600–1400			61
TiO_2_ (rutile)		1655		1595		1545, 1430		32
α-Fe_2_O_3_		1665	1560	1590				35
γ-Al_2_O_3_		1688		1613				33
TiO_2_		1666		1602				34
MgO_2_	1710	1686, 1675		1626			1580	36

Acetone								
SiO_2_	1709							31
TiO_2_ (rutile)		1680						32
MgO, NiO						1560–1510		62
α-Fe_2_O_3_		1685, 1675					1540	35
γ-Al_2_O_3_		1696					1596	33
γ-Al_2_O_3_	1705–1700	1702–1690						37 and 60
TiO_2_		1702						34

Unfortunately, it was impossible to acquire any meaningful spectra in the case of 8.7%_wt_ Au/CeO_2_ catalyst. The catalyst has deep purple colour and the thin pellets made of its powder were either too fragile to withstand the mounting procedure in the quartz holder or they were completely not transparent for the IR laser light, thus, almost no beam intensity could be registered by the detector in whole energy range regardless of the spectrometer configuration and settings.

Similarly, the pure TiO_2_ powder allowed preparing pellets that never survived beyond the MesOx adsorption phase, so no spectrum was acquired for H_2_-influenced MesOx adsorbed on TiO_2_, even though numerous attempts were embarked.

Although the IR spectra were acquired from 4000 cm^−1^ to 1300 cm^−1^ the interesting bands responsible for C–O and C–C bonds vibrations as well as –CH_3_ bending modes and *δ*_CH_ deformation vibration of hydrocarbon groups can be found in the range between 1800–1300 cm^−1^. Above 3000 cm^−1^ all bands characteristic for MesOx and its derivatives overlay with the very broad and intense bands corresponding to –OH vibrations. The ^sp^2^^C–H and ^sp^3^^C–H vibration bands in the 3000–2900 cm^−1^ energy range do not provide clear conclusions about neither MesOx adsorption state nor interaction with H_2_, because mainly only the intensity of the bands change. Hence, these mentioned energy ranges are excluded from the discussion presented below.

**Fig. 2:**
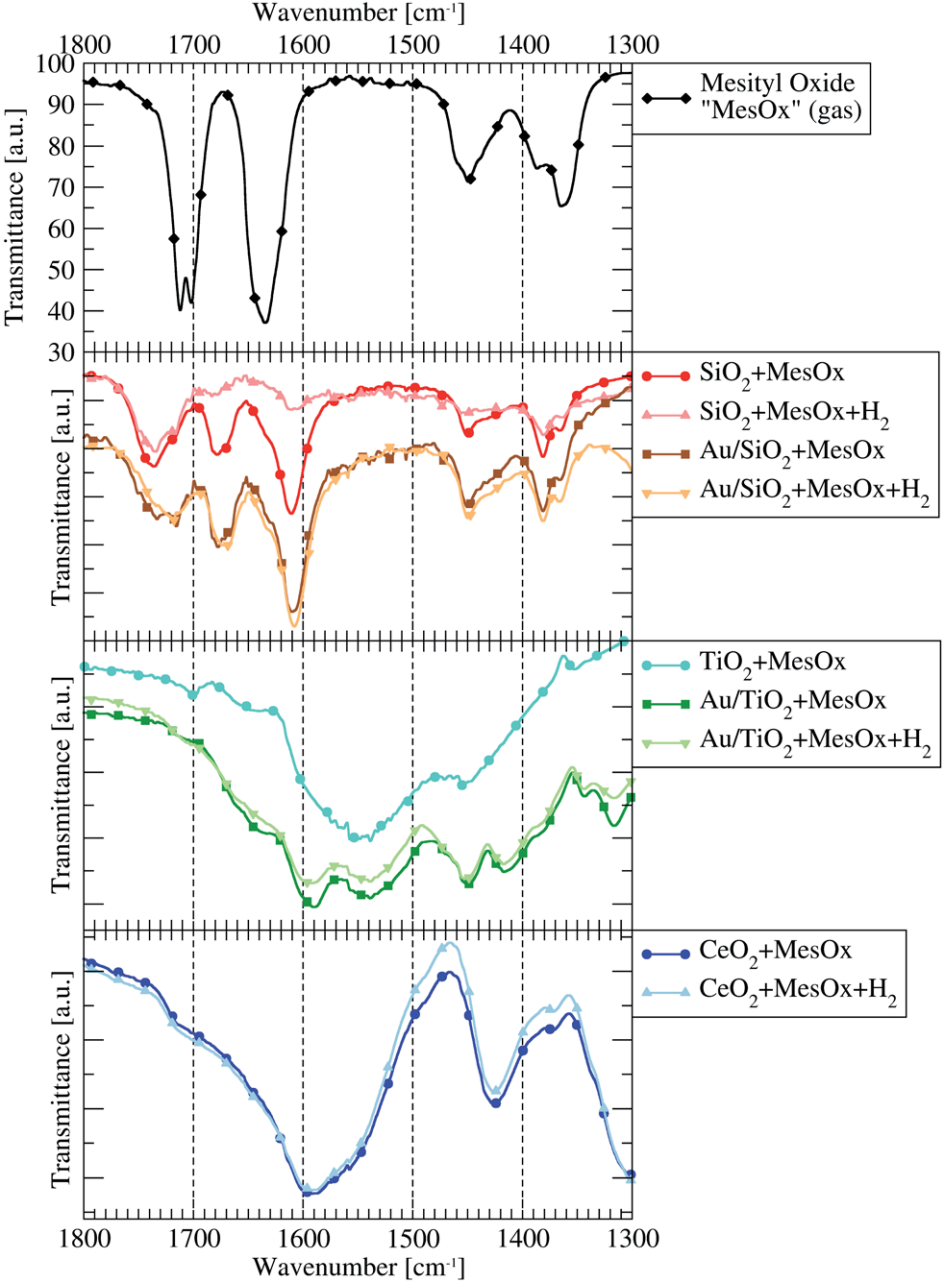
The IR spectra limited to the 1800–1300 cm^−1^ energy range presenting the vibrational bands of free gaseous mesityl oxide (MesOx, black line on the top plot), its derivative species (before and after introduction of H_2_ subsequently evacuated from the system) adsorbed on the surface of catalysts (Au/material + MesOx) or pure supports (not containing gold nanoparticles; material + MesOx).

The spectrum of pure MesOx in the gas phase (vapour pressure equalled saturation at room temperature), presented in Fig. 2, exhibits sharp bands in the 1800–1300 cm^−1^ range and a couple of overlaying bands of weaker intensity in the 3000–2900 cm^−1^ range. The latter correspond to stretching vibrations of the ^sp^2^^C–H and ^sp^3^^C–H bonds. The former, between 1500 cm^−1^ and 1300 cm^−1^, originate from the methyl groups bending modes and deformation vibration of hydrocarbon bonds.^31–34^ Additionally, between 1800 cm^−1^ and 1600 cm^−1^, there are signals connected to stretching vibrations of the carbonyl group (1702 cm^−1^ and 1712 cm^−1^) and to stretching vibration of the ^α^C=^β^C double bond.

**Fig. 3:**
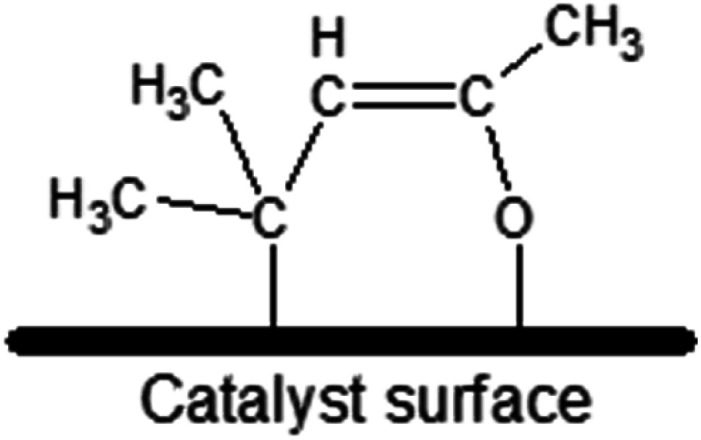
Possible schematic representation of the enolate adsorption form (also called “metallacycle” by ref. 40) of mesityl oxide on the catalyst surface – adsorption occurs thanks to binding to the surface of the oxygen atom from the carbonyl group and the β-carbon atom with respect to the carbonyl group.

The most chemically active catalyst from the studied set was the one based on CeO_2_ support. The dominant form of adsorbed MesOx found on the bare CeO_2_ surface not decorated with AuNPs is the enolate form (*i.e.* adsorption through the oxygen atom and the β-carbon atom, Fig. 3) as evidenced by band peak-top at 1589 cm^−1^ before and 1590 cm^−1^ after introduction with H_2_ (ref. 33, 35 and 36, Fig. 2). Additionally, the right-hand shoulder ranging till 1500 cm^−1^ suggests that adsorption of MesOx yielded species containing carboxylic groups^33^ which could be formed only by adsorption supported by oxygen atoms or hydroxyl groups of the materials surface. The small hump observed at 1714 cm^−1^ suggests that also adsorption through carbonyl group to the Brønsted acidic centre occurs.^36,37^ With regard to observation of the dominant enolate form, the spectrum profile for wavenumbers smaller than 1500 cm^−1^ changes significantly, reflecting new vibrational constants of bending of the –CH_3_ groups neighbouring the β-carbon atom by forming two bands centred around 1371 cm^−1^ and 1423 cm^−1^^37^

Especially the enolate form, but also the carboxylic one, are expected to be very active species due to serious relocation of the electron density across the adsorbed molecule. Thus, adsorption of MesOx in these forms on the surface supposedly favours hydrogenation of: (1) ^α^C=^β^C double bond to saturated ketone (what was clearly observed by GC and MS); (2) C=O carbonyl group to allyl alcohol (only traces of AA were detected by MS); or (3) regrouping of double bonds upon desorption without hydrogenation to create iso-mesityl oxide (4-methylpentan-4-en-2-one).

TiO_2_ alone has clear preference to support MesOx adsorption by incorporation of surface oxygen atoms as evidenced by the carboxylic group vibrational band centred at approx. 1550 cm^−1^ (ref. 33, Fig. 2). Additionally, the two bands at 1701 cm^−1^ and 1642 cm^−1^ point to possible adsorption of the MesOx carbonyl group to acidic centres, Broensted and Lewis type respectively,^36,37^ present on the TiO_2_ surface. The small acidity of TiO_2_ was utilised during the Au/TiO_2_ catalyst synthesis to attach Au(OH)_*x*_^3−*x*^ complex ions to the oxide surface through ion exchange. In the 1500–1300 cm^−1^ range, only a C–H deformation band at 1454 cm^−1^ can be observed while methyl group bending modes remain invisible.

On the contrary to pure TiO_2_, the Au-decorated surface of titania shows ability to stabilise the adsorbed MesOx in the enolate form due to which a band at 1588 cm^−1^ can be spotted. Correspondingly to the formation of enol-type species, bands at wavenumbers below 1450 cm^−1^ are observed similarly to the case of pure CeO_2_. Au/TiO_2_ spectra still exhibit features characteristic for pure TiO_2_, *i.e.* interaction of the MesOx carbonyl group with acidic centres of the surface (1708 cm^−1^ and 1654 cm^−1^) and with surface oxygen atoms taking part in carboxyl group formation (1539 cm^−1^).

Samples containing SiO_2_ yield much different IR spectra demonstrating basically 3 types of MesOx adsorption to acidic sites: the H-bonded (Broensted site) and electron-donating (Lewis site) carbonyl group corresponding to 1735 cm^−1^ and 1668–1678 cm^−1^ bands, respectively; and the C=C double bond adsorbed also to a Lewis site as indicated by the 1607–1611 cm^−1^ band.

Pure silica was unable to hold MesOx on its surface after H_2_ was introduced to and evacuated from the system. On the other hand, the deposition of gold helped keeping the MesOx on the surface of the catalyst under H_2_ atmosphere (Fig. 2) and, furthermore, the bands observed at 1609 cm^−1^, 1677 cm^−1^ and 1732 cm^−1^ shift to lower wavenumbers by 2–14 cm^−1^. The change of strength of the carbonyl group and ^α^C=^β^C double bonds may be interpreted in terms of ability of ultrasmall (<5 nm) AuNPs to interact with hydrogen^38,39^ and supplying its active forms to the adsorbed MesOx. At the same time, the bands connected to vibration of methyl groups remain unchanged. They closely resemble the band structure of gaseous MesOx what is expected with regard to adsorption of only the conjugated bonds on the acidic sites.

Pure TiO_2_, as well as other pristine supports, shows no catalytic activity towards neither hydrogenation nor isomerisation of MesOx at temperatures up to 350 °C, but the Au-containing systems demonstrate clear conversion. Additionally, comparing Au/CeO_2_ and Au/TiO_2_ results, the conclusion can be drawn that, in terms of hydrogenation of MesOx to SK and, possibly, also other derivatives under optimal conditions, the enol-type of adsorption of MesOx would be favourable over adsorption leading to formation of a carboxyl group. The case of Au/SiO_2_ catalyst adds also the observation that weakening of the C=C double bond through its adsorption to a Lewis acidic site is sufficient for its hydrogenation provided that the system can supply active forms of hydrogen (as gold-decorated supports do). Although the 1609–1607 cm^−1^ band corresponding to C=C adsorption is most prominent in the SiO_2_-based systems, such adsorption type cannot be ruled out in the cases of other catalytic systems.

In general, in this work the term “Lewis acidic site” covers broad range of species that are electrophilic, just to mention: oxygen vacancies present in the catalysts' oxide supports, and gold which, as a transition metal, has its d-band in principle able to accept/exchange electrons with C=C or C=O bonds.^40,41^

### Temperature-programmed reduction

The TPR studies on CeO_2_ and Au/CeO_2_ have been once published by us elsewhere.^42^ In brief, we proved that Au/CeO_2_ catalyst, unlike pure CeO_2_, is able to cyclically adsorb and immediately release hydrogen in temperatures below approx. 250 °C. Attention was also put on the capability of ceria itself to adsorb carbon dioxide from air. The physically bound CO_2_ can be desorbed below 150 °C, but only above 300 °C the strongly adsorbed CO_2_ is released after just thermally induced decomposition to CO (2 peaks at 325 °C and 670 °C proved by MS analysis). As the catalyst contains AuNPs facilitating adsorption of hydrogen, different H_*x*_CO_*y*_ species are presumably formed.^43–45^ Hence, carbon oxides are released from the Au/CeO_2_ surface at higher temperatures as evidenced by peaks shifted to 484 °C and 805 °C.

The TPR profiles of pure and Au-decorated CeO_2_ are, naturally, the sum of CO_2_ desorption-related phenomena as well as phase reduction of ceria. The latter starts at the surface, judging by the peak at 520 °C, to be followed by bulk reduction represented by the peak at 880 °C.^44^ The Au/CeO_2_ catalyst shows similar behaviour, although H_2_ consumption for surface reduction starts as early as from 300 °C and the bulk transformation yields peak at 810 °C.

Comparing to materials containing CeO_2_, the TPR profiles of the other materials show peaks that are approx. an order of magnitude smaller (considering both peak height and area) while similar masses of samples were used. Thus, the marked capability of CeO_2_ to interact with and store hydrogen (*e.g.* [ref. 46]) necessary to reduce MesOx is confirmed.

**Fig. 4:**
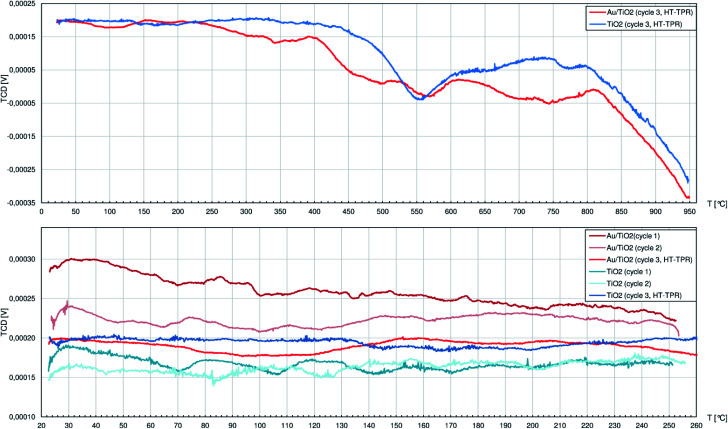
The profiles registered during temperature-programmed reduction (TPR) studies by the thermal-conductivity detector (TCD) of the 0.5%_wt_ Au/TiO_2_ catalyst and pure TiO_2_. There were 2 cycles (cycles no. 1 and 2) of temperature ramp up to 250 °C and a 3rd cycle (cycle 3, HT-TPR) up to 950 °C. Specimens were fed with the 10%_vol_ H_2_/Ar mixture.

Below 400 °C the TPR profile of pure TiO_2_ (Fig. 4) shows only peaks with negligible height: during the 1st cycle at 71 °C, 107 °C, 143 °C and 171 °C and during the 2nd cycle only 3 peaks at 84 °C, 125 °C and 202 °C. Unlike CeO_2_, titania does not adsorb large quantities of air components, so at relatively low temperatures only little amounts of thermally desorbed gases were released to the H_2_/Ar stream slightly diluting it. The first strong peak is observed in the 3rd cycle at 554 °C and corresponds to slow release of oxygen ions as H_2_O from the rutile structure caused by H_2_ adsorbed on the oxygen vacancies V_O_^++^.^47^ The more V_O_^++^ are formed, the more O^2−^ anions diffuse outwards from the inner titania structure, and the process seems to behave like an autocatalytic one. While the reduction proceeds smoothly between approx. 600 °C and 800 °C, it is followed by heavy increase of hydrogen consumption that is interrupted at 950 °C after the TPR system reaches its technological limits. Above approx. 750 °C anatase is expected to transform to rutile^48^ which easily undergoes the same mechanism as described above, adding its contribution to the TPR curve.

Similarly to pure TiO_2_, the Au/TiO_2_ catalyst can be considered as rather not influenced by hydrogen atmosphere up to 210 °C when the H_2_ consumption starts to increase until the first peak top at 342 °C. Later above 400 °C, numerous peaks are identified, namely at: 456 °C, 496 °C, 569 °C, 700°, 745 °C and above 810 °C the reduction process largely consuming H_2_ could continue well above 950 °C.

AuNPs on the TiO_2_ clearly facilitate its interaction with hydrogen. The reduction passes through more stages that also happen at approx. 150 °C lower temperatures comparing to pure titania. The improved affinity of Au/TiO_2_ system to H_2_, like the augmented adsorption of MesOx on Lewis sites, can both rely on better electron-accepting properties of TiO_2_ in the proximity of AuNPs.

**Fig. 5:**
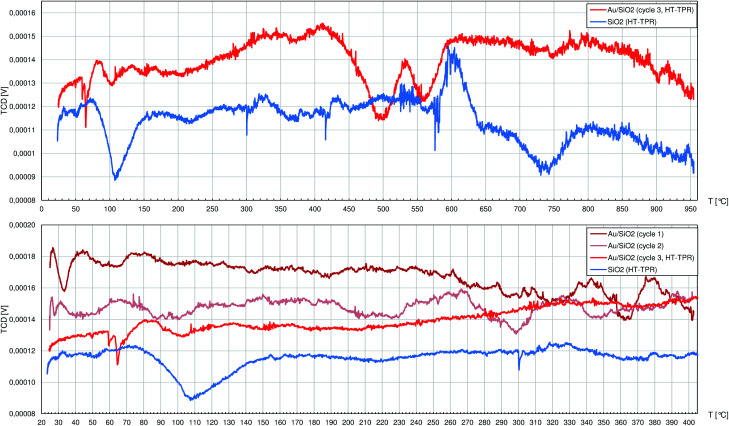
The profiles registered during temperature-programmed reduction (TPR) studies by the thermal-conductivity detector (TCD) of the 10.0%_wt_ Au/SiO_2_ catalyst and pure SiO_2_. In the case of the catalyst, which first underwent the activation process (see the Experimental section), there were 2 cycles (cycles no. 1 and 2) of temperature ramp up to 400 °C and a 3rd cycle (cycle 3, HT-TPR) up to 950 °C. Pure SiO_2_ was subjected only to one cycle up to 950 °C. Specimens were fed with the 10%_vol_ H_2_/Ar mixture.

SiO_2_ allowed to collect a very noisy but flat TPR profile (Fig. 5) with only 3 peaks at 108 °C, 604 °C and 737 °C featured by poor signal-to-noise ratio. Pure SiO_2_ during heating, even under H_2_ atmosphere, can release mainly adsorbed water (not visible by TCD detector due to cold trap mounted in the tubing) and residual adsorbed air components, but the observed peaks are of little importance to the R-(V)ABUCC reaction.

The TPR experiment on the Au/SiO_2_ catalyst had to be preceded by the post-synthesis activation process (decomposition of gold–ethylenediamine complex with crystallisation of AuNCrs followed by burning of the organics residuals) performed *in situ*. Thus, the 1st and 2nd cycles finished at 400 °C regarding the temperature reached in the last stage of activation. Any peaks that can be eventually spotted in these profiles were insignificant (*i.e.* they can not be clearly described because of comparably high alternations of the baseline). During the 3rd cycle, 2 peaks between 450 °C and 600 °C are noted. On the absolute scale they have to be regarded as weak, but there are no corresponding peaks in the TPR profile of pure SiO_2_. Indeed, the observed in this case H_2_ consumption is minute and occurs beyond the catalyst working conditions for R-(V)ABUCC reaction, hence it is excluded from further analysis.

### Morphology and crystal structure

**Fig. 6:**
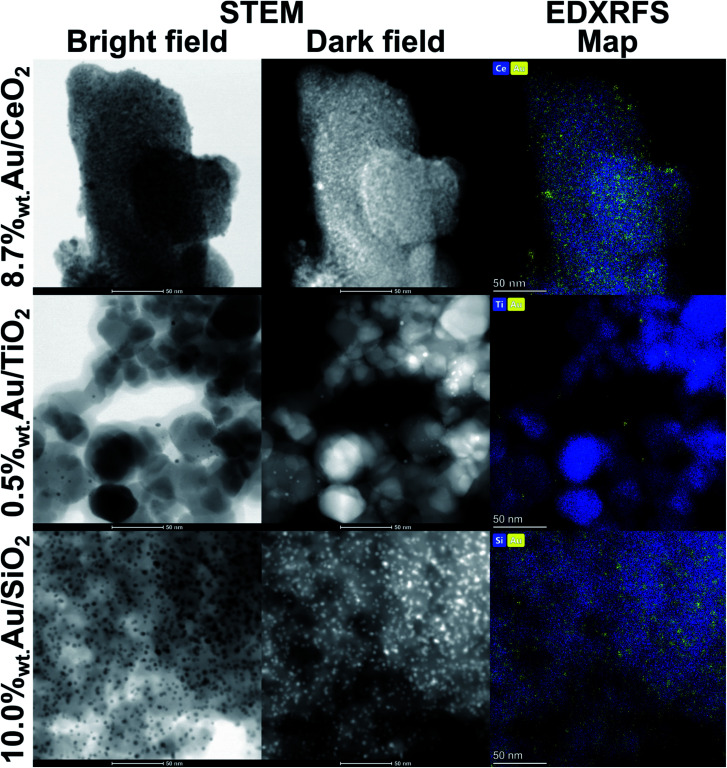
STEM bright and dark field images accompanied by the corresponding EDXRFS maps presenting the morphologies of 8.7%_wt_ Au/CeO_2_, 0.5%_wt_ Au/TiO_2_ and 10.0%_wt_ Au/SiO_2_ catalysts while differentiation between AuNPs and support is facilitated by composition maps limited to show distribution of only Au, Ce, Ti and Si elements.

Transmission electron microscopy (TEM) was used in the scanning mode (STEM) to investigate the morphology and elemental composition (by means of energy-dispersive X-ray fluorescence spectroscopy, EDXRFS) of the catalysts (Fig. 6).

AuNPs deposited on SiO_2_ have narrow size distribution and virtually all observed particles are smaller than 5 nm. The PXRD diffraction data provides estimation of the volume-weighted crystallite size around 3.5 nm (Table 2). AuNPs are also uniformly dispersed on the SiO_2_ surface. This feature has to be attributed to the synthesis method based on the immobilisation of Au^3+^–ethylenediamine complex on the catalyst support. Thermal decomposition of the AuNPs precursor assisted by simultaneous reduction of gold ions to metallic form by H_2_ provides instant seeding of AuNPs in the whole volume of the prospective catalyst. Thus, growth of bigger crystals at the expense of formation of numerous smaller ones is inhibited.

**Table 2:** Structural parameters of the phases identified in the diffraction patterns of the 8.7%_wt_ Au/CeO_2_, 0.5%_wt_ Au/TiO_2_ and 10%_wt_ Au/SiO_2_ catalysts. The last figure regression error is given in parenthesis

Material	Crystal phase	Index	Lattice parameters		%_wt_^a^	Size^b^ [nm]	Strain–stress (*ε*) factor
			*a* [Å]	*c* [Å]			
8.7%_wt_ Au/CeO_2_	CeO_2_	1	5.4202(1)	—	56.1(4)	16.0(4)	0.00111(2)
		2	5.4229(3)	—	27.4(4)	6.5(2)	0.00391(11)
	Au		4.0823(2)	—	16.4(1)	23.0(20)	0.0020(10)

0.5%_wt_ Au/TiO_2_	TiO_2_ (anatase)	1	3.7908(2)	9.5228(7)	61.7(40)	20.6(10)	0.00045(3)
		2	3.7935(5)	9.499(4)	24.5(40)	10.7(7)	0.00186(16)
	TiO_2_ (rutile)		4.6015(3)	2.9632(2)	13.6(2)	27.2(10)	0.00(6)
	Au		4.0780^c^	—	0.2(40)	4.6(17)	n/a^d^

10.0%_wt_ Au/SiO_2_	Au		4.0776(45)	—	100.0 e	3.5(12)	0.01(2)

a According to Rietveld refinement based on the unit crystal cell mass.

b Volume-weighted average crystallite size calculated according to Laue^59^ from integral breadth of the fitted Voigt function.

c The lattice parameter *a* of gold was constrained to the reference value,^50^ because the amount of gold (0.5%_wt_) was too small to yield a stable fit of the model phase to the experimental data.

d The scattering contribution of gold to the experimental line profile was too small to allow differentiation between the peak broadening originating from size and strain–stress, hence the integral breadth was attributed wholly to size-related effects.

e The scattering contribution of amorphous SiO_2_ has not been assessed in terms of silica content.

Application of ethylenediamine as the Au^3+^ ligand was not possible in syntheses using other supports due to insufficient binding of the AuNPs precursor to the CeO_2_ and TiO_2_ surface. For these supports, the ion exchange of hydroxyl groups between the oxide surface and Au(OH)_4_^−^ was utilised. Although the method can be considered efficient for synthesis of Au/CeO_2_ catalyst, limited success was observed for Au/TiO_2_ system. The Au loading on TiO_2_ surface was limited to 0.5%_wt_, but small AuNPs were obtained, similarly to the SiO_2_-based catalyst. That small content of Au prevented fully free fitting of the diffraction profile and the *a* lattice parameter of gold had to be constrained to ideal 4.078 Å.

Commercially available TiO_2_ under the name “Aeroxide” consists of 2 titania phases: anatase and rutile. The diffraction peaks exhibit marked broadening of the peaks' bases which resulted in using 2 fractions of anatase phase to fit the experimental scattering profile. The lattice parameters *a* and *c* of the phases differ by only 0.3 pm, but the average-by-volume crystallite size varies by the factor of 2, namely the diameters are established around 20 and 10 nm (Table 2).

The Au/CeO_2_ catalyst contains AuNPs with broad distribution of sizes, although their desired uniform dispersion is maintained as in the cases of other catalysts. The overview presented in Fig. 6 shows that the majority of AuNPs is smaller than 10 nm, *i.e.* that fraction does not differ much from gold crystals deposited on other supports. However, occasionally larger particles can be spotted which is an important factor in the diffraction patterns analysis. Diffraction is a phenomenon that heavily depends on the volume of the coherently scattering object, yet occurrence of large crystals shifts the volume-weighted average crystallite size to above 20 nm (Table 2).

The established CeO_2_ and Au lattice parameters were found to differ by only approx. 0.5–1 pm from the reference values: 5.411 Å and 4.078 Å, respectively.^49^ The slight expansion of CeO_2_ structure could be explained by the deviation of the stoichiometry. CeO_2–*x*_ (as better to be referred to), especially the nanocrystalline powder, was reported to expand the volume of the unit cell with loosing oxygen ions due to larger Ce^3+^ ionic radius that even compensates the formation of oxygen vacancies.^50^ The issue has been also covered by us elsewhere.^42^ The increase of Au lattice parameter resulted from the residual intensity between the 1 1 1 and 2 0 0 diffraction peaks not included in the Au fcc crystal model. This additional scattering contribution was characteristic for multiply-twinned particles.^51–53^ When 5 tetrahedra crystallites form one particle, that particle has 5-fold symmetry (a, possibly truncated, decahedron is obtained) along [110] direction, but the crystal lattices of the building crystallites have to be strained in order to connect all the crystallites boundaries.^54^ This strain has to be applied mainly to the (111) and (200) planes (parallel to [110] direction) what leads to decreasing the 2*θ* angle difference between the 1 1 1 and 2 0 0 peaks, hence the fcc model always leaves the residual not described intensity in the diffraction pattern.

In general, decahedral nanoparticles in TEM images resembled a much more common fcc crystals ordering and only when aligned with the [110] direction perpendicular to the imaging plane it might be possible to see the twinned single-crystal domains wrapped around the 5-fold symmetry axis.

## Conclusions

Our research results showed the possibility of investigating the adsorbed forms of reactants on the surface of heterogeneous powder catalyst and connecting the observed catalytic activity with the way the material interacts with hydrogen, thus allowing reduction of organic compounds. To achieve our goal, we employed *in situ* infrared spectroscopy (IR) in transmission mode, temperature-programmed reduction (TPR) studies as well as electron microscopy (TEM) and X-ray diffraction (PXRD) for morphology and crystals structure determination.

Presence of gold nanoparticles was proved to be essential when they are supported on the selected for this research oxides for the reduction of α,β-unsaturated carbonyl compounds. As proved by the IR spectra, mesityl oxide (MesOx) adsorbs on pure oxides but remains insensitive to hydrogen atmosphere, Au seems to us to be involved mainly in adsorption and activation of H_2_ molecule. Hence, increased hydrogen consumption in TPR studies was observed at lower temperatures than for pure ceria, titania and silica. Immediately reversible after gas atmosphere change binding of hydrogen by Au/CeO_2_ system was spotted even below 100 °C. Both TiO_2_- and SiO_2_-based catalysts additionally benefited from their surface decoration with Au. In the former case, enolate derivatives of MesOx were found adsorbed on the surface on the contrary to observations made for pure TiO_2_. The latter example revealed the direct interaction between MesOx-related species and Au evidenced by more stable and longer-lasting adsorption of MesOx on the Au/SiO_2_ catalyst surface.

We have identified 3 most desirable adsorption forms of MesOx on the catalysts surface that allow reduction of MesOx, in our research to a saturated ketone (namely 4-methylpentan-2-one, also called methyl-isobutyl ketone), that is:

• Adsorption on CeO_2_ and TiO_2_ through the oxygen atom from the carbonyl group and the β-carbon atom with respect to the original carbonyl group;

• Adsorption leading to formation of species containing a carboxyl group formed by incorporation of the hydroxyl group available on the CeO_2_ and TiO_2_ surfaces;

• Adsorption of the ^α^C=^β^C double bond to a Lewis acidic site that weakens the conjugation of this double bond with the double bond in the neighbouring carboxyl group.

Activation of carbonyl group seems to depend on the properties of the oxide support. Namely, the more reducible and electrophilic the oxide support is, the more likely it is that mesityl oxide adsorption incorporates carbonyl group so that highly activated intermediate products presumably easily interacting with active hydrogen moieties are formed.

The overall observed catalytic reaction output may be different from what is suggested by favoured adsorbed intermediate products.^55^ According to Loffreda and co-workers^56,57^ some final products may not be obtained, at least not in large quantities, due to their desorption from the catalyst surface being the limiting step. Hindered desorption may, thus, allow achieving other compounds with good yields.

Mesityl oxide was chosen as the model compound due to being the simplest α,β-unsaturated ketone with similar steric hindrance around both C=C and C=O bonds, thus creating no bias on hydrogenation pathway. The choice of the organic compound and the catalyst determines hydrogenation product and reaction pathway. Hence, adsorption of prenal (3-methylbut-2-enal) on Pt (111) also involved formation of enol-type species,^55^ but use of crotonaldehyde, furfural and cinnamaldehyde with NiBi intermetallic compound revealed adsorption of only C=O group, whilst 2 bands at 1650–1550 cm^−1^ characteristic for “free state” C=C were observed at the same time.^8^ Others suggest significant role of keto–enol tautomerisation in hydrogenation mechanism (studied on the example of acetophenone), as the tautomer can be stabilised by another molecule being the ketone.^58^

Our next step is to perform *in operando* PXRD studies focused on relating the crystal structure changes of the solid powder heterogeneous catalyst (all crystal phases) with its chemical activity. The research we have presented in this work is vital for understanding the catalytic properties of the selected systems to later interpret properly the crystal structure dynamics under *in operando* conditions. Some of the reaction conditions were deliberately not optimised because the PXRD system that we are commissioning after adopting it to work with vapours of liquids imposes slight limitations on how the chemical reaction can be carried out. The crucial condition is the reagents and products to be in the gas phase.

## Author contributions

MZ – conceptualisation, data curation, formal analysis, funding acquisition, investigation, methodology, project administration, resources, supervision, validation, visualisation, writing – original draft, writing – review & editing.

WJ – investigation, validation, resources, writing – review & editing.

ZK – formal analysis, funding acquisition, supervision, writing – review & editing.

## Conflicts of interest

There are no conflicts to declare.

## Supplementary Material

RA-012-D1RA09434C-s001

## References

[cit1] Guzman J., Gates B. C. (2003). Angew. Chem..

[cit2] Zhang X., Shi H., Xu B. (2005). Angew. Chem..

[cit3] Zhang X., LlabresiXamena F. X., Corma A. (2009). J. Catal..

[cit4] Xue X., Niu M., Xu Y., Wang Y. (2017). RSC Adv..

[cit5] Gizinski D., Goszewska I., Zielinski M., Lisovytskiy D., Nikiforov K., Masternak J., Zienkiewicz-Machnik M., Srebowata A. (2017). Catal. Commun..

[cit6] Zeyang L., Yongjie W., Kaihong L., Shanshan W., Haocheng L., Yuanli Z., Baoming H., Chunxia T., Guohua L. (2021). Front. Chem..

[cit7] Cao Y., Guerrero-Sańchez J., Lee I., Zhou X., Takeuchi N., Zaera F. (2020). ACS Catal..

[cit8] Yu J., Yang Y., Chen L., Li Z., Liu W., Xu E., Zhang Y., Hong S., Zhang X., Wei M. (2020). Appl. Catal., B.

[cit9] Wang K., Yang B. (2017). Catal. Sci. Technol..

[cit10] Kuai L., Chen Z., Liu S., Kan E., Yu N., Ren Y., Fang C., Li X., Li Y., Geng B. (2020). Nat. Commun..

[cit11] Huang X., Jones T., Fedorov A., Farra R., Coperet C., Schlogl R., Willinger M.-G. (2021). Adv. Mater..

[cit12] Kaszkur Z., Rzeszotarski P., Juszczyk W. (2014). J. Appl. Crystallogr..

[cit13] Kaszkur Z., Zielinski M., Juszczyk W. (2017). J. Appl. Crystallogr..

[cit14] Gellman A. J., Tysoe W. T., Zaera F. (2015). Catal. Lett..

[cit15] User manuals of printers manufactured by Brother (TM)

[cit16] Wawrzenczyk C., Wisinska K. (2007). Pol. J. Chem..

[cit17] Tysoe W. T. and Roy S. P., Enantioselective reduction of ketones on clean and chirally modified single crystal model Pd and Pt catalysts, in Abstracts of Papers, 262nd ACS National Meeting & Exposition, Atlanta, GA, United States, 2021

[cit18] Ciriminna R., Pagliaro M., Falletta E., Della C. P., Teles J. H. (2016). Angew. Chem., Int. Ed..

[cit19] Ishida T., Koga H., Okumura M., Haruta M. (2016). Chem. Rec..

[cit20] Liu X., Liu M.-H., Luo Y.-C., Mou C.-Y., Lin S. D., Cheng H., Chen J.-M., Lee J.-F., Lin T.-S. (2012). J. Am. Chem. Soc..

[cit21] Saqlain M. A., Hussain A., Siddiq M., Leitao A. A. (2016). Appl. Catal., A.

[cit22] Wang Y.-G., Cantu D. C., Lee M.-S., Li J., Glezakou V.-A., Rousseau R. (2016). J. Am. Chem. Soc..

[cit23] Haruta M. (2002). CATTECH.

[cit24] Zielinski M. , Master thesis Warszawa, Warsaw University of Technology, Faculty of Chemistry, Poland, 2015, https://apd.usos.pw.edu.pl/diplomas/4922/

[cit25] Lam Y. L., Boudart M. (1977). J. Catal..

[cit26] Block B. P., C Bailar J. (1951). J. Am. Chem. Soc..

[cit27] Louw W. J., Robb W. (1969). Inorg. Chim. Acta.

[cit28] Kitada K. , inventor, US Pat., 6087516, 2000

[cit29] Merck KGaA , Material Safety Data Sheet: Mesityl oxide for synthesis Darmstadt, Germany, 2015

[cit30] Fisher Scientific – Acros Organics , Material Safety Data Sheet: 4-methyl-2-pentanol Fair Lawn, NJ, USA; 2018

[cit31] Young R. P., Sheppard N. (1967). J. Catal..

[cit32] Griffiths D. M., Rochester C. H. (1978). J. Chem. Soc., Faraday Trans..

[cit33] Hanson B. E., Wieserman L. F., Wagner G. W., Kaufman R. A. (1987). Langmuir.

[cit34] El-Mazawi M., Finken A. N., Nair A. B., Grassian V. H. (2000). J. Catal..

[cit35] Busca G., Lorenzelli V. (1982). J. Chem. Soc., Faraday Trans..

[cit36] Braun F., Di Cosimo J. I. (2006). Catal. Today.

[cit37] Panov A., Fripiat J. J. (1998). Langmuir.

[cit38] Silverwood I. P., Rogers S. M., Callear S. K., Parker S. F., Catlow C. R. A. (2016). Chem. Commun..

[cit39] Watkins W. L., Borensztein Y. (2017). Phys. Chem. Chem. Phys..

[cit40] Delbecq F., Sautet P. (1995). J. Catal..

[cit41] Lan X., Wang T. (2020). ACS Catal..

[cit42] Zielinski M., Juszczyk W., Sobczak J. W., Kaszkur Z. (2022). Phys. Chem. Chem. Phys..

[cit43] Binet C., Badri A., Boutonnet-Kizling M., Lavalley J.-C. (1994). J. Chem. Soc., Faraday Trans..

[cit44] Araiza D. G., Gomez-Cortes A., Diaz G. (2017). Catal. Sci. Technol..

[cit45] Schilling C., Ziemba M., Hess C., Ganduglia-Pirovano V. (2020). J. Catal..

[cit46] Morgensen M., Sammes N. M., Tompsett G. A. (2000). Solid State Ionics.

[cit47] Khader M. M., Kheiri F. M. N., El-Anadouli B. E., Ateya B. G. (1993). J. Phys. Chem..

[cit48] Murray J. L., Wriedt H. A. (1987). Bull. Alloy Phase Diagrams.

[cit49] Zielinski M. , PhD Thesis, Institute of Physical Chemistry, Polish Academy of Sciences, Warszawa, Poland, 2019, https://rcin.org.pl/dlibra/publication/edition/81875?id=81875

[cit50] Wyckoff R. W. G. , Crystal structures, Interscience Publishers, New York, London, Sydney, 2nd edn, 1963

[cit51] Hofmeister H. (2003). Fivefold Twinned Nanoarticles. Encycl. Nanosci. Nanotechnol..

[cit52] Barnard A. S., Young N. P., Kirkland A. I., van Huis M. A., Xu H. (2009). ACS Nano.

[cit53] Kirkland A. I., Jefferson D. A., Tang D., Edwards P. P. (1991). Proc. R. Soc. London, Ser. A.

[cit54] Ling C., Yu Y. (2019). IUCrJ.

[cit55] Kliewer C. J., Bieri M., Somorjai G. A. (2009). J. Am. Chem. Soc..

[cit56] Loffreda D., Delbecq F., Vigne F., Sautet P. (2006). J. Am. Chem. Soc..

[cit57] Loffreda D., Delbecq F., Vigne F., Sautet P. (2005). Angew. Chem., Int. Ed..

[cit58] Attia S., Schmidt M. C., Schoeder C., Weber J., Baumann A.-K., Schauermann S. (2019). J. Phys. Chem. C.

[cit59] von Laue M. (1926). Ann. Phys..

[cit60] Panov A. G., Fripiat J. J. (1998). J. Catal..

[cit61] Griffiths D. M., Rochester C. H. (1977). J. Chem. Soc., Faraday Trans..

[cit62] Miyata H., Toda Y., Kubokawa Y. (1974). J. Catal..

[cit63] Bonarowska M., Wojciechowska M., Zielinski M., Kiderys A., Zielinski M., Winiarek P., Karpinski Z. (2016). Molecules.

[cit64] Benbenek S., Fedorynska E., Winiarek P. (1993). React. Kinet. Catal. Lett..

[cit65] Zielinska P. , Master Thesis Warszawa, Warszaw University of Technology, Faculty of Chemistry, Poland, 2015, https://repo.pw.edu.pl/info/master/WUT992802c1f859492bb1dbed999a309fae/

