# Supplementary material for: Studies of adsorption of α,β-unsaturated carbonyl compounds on heterogeneous Au/CeO2, Au/TiO2 and Au/SiO2 catalysts during reduction by hydrogen
Source: RSC Adv. 2022 Feb 15;12(9):5312–23. doi: 10.1039/d1ra09434c (PMC8981266; doi:10.1039/d1ra09434c)
Supplement: RA-012-D1RA09434C-s001 [file RA-012-D1RA09434C-s001.pdf]

Electronic Supplementary Material (ESI) for RSC Advances.
This journal is © The Royal Society of Chemistry 2022

Electronic Supplementary Information

to article entitled"

# "Studies of adsorption of α,β–unsaturated carbonyl compounds on heterogeneous Au/$CeO_2$, Au/$TiO_2$ and Au/$SiO_2$ catalysts during reduction by hydrogen"

by

Maciej Zielinski,*[a] Wojciech Juszczyk [a] and Zbigniew Kaszkur [a]

[a] Institute of Physical Chemistry, Polish Academy of Sciences, ul. M. Kasprzaka 44/52, Warszawa, Poland

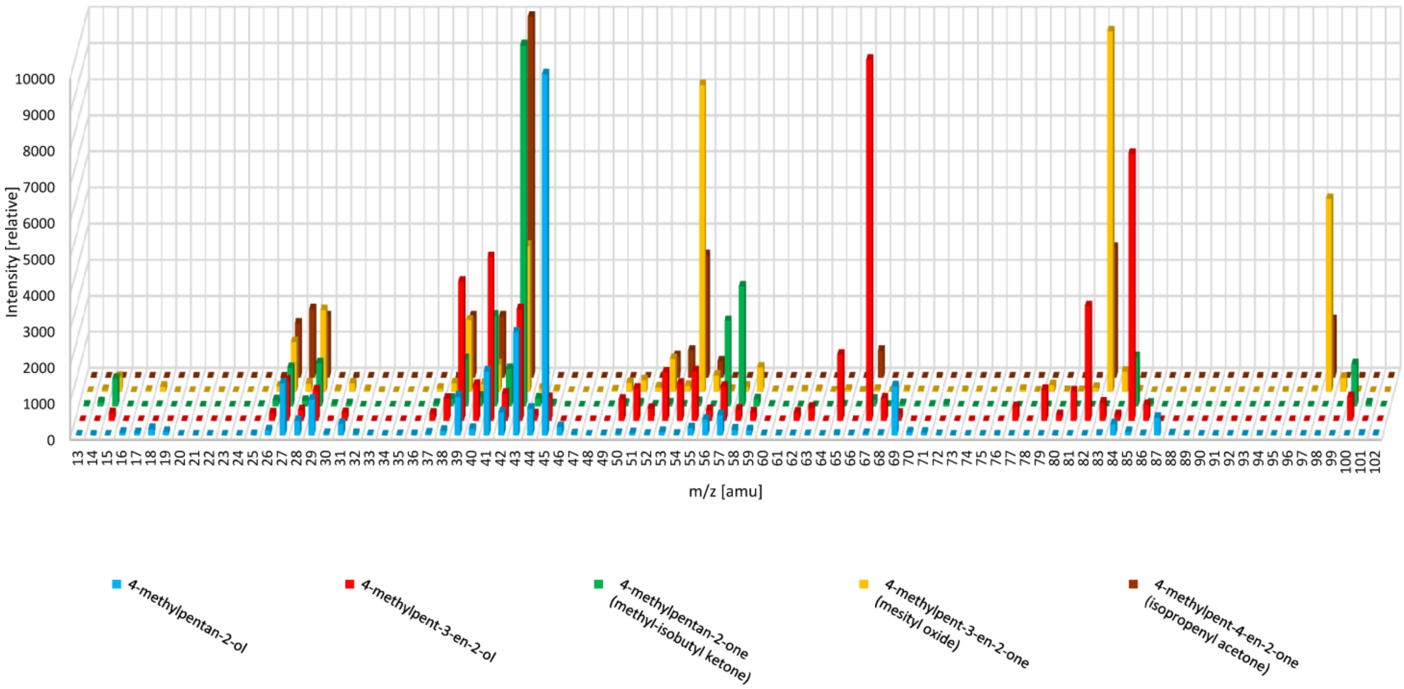


**Figure S 1.** The bar chart presenting the reference mass spectrometry signals (atomic mass units [amu] divided by the ion charge, m/z) and their intensities of mesityl oxide (4-methylpent-3-en-2-one) and its hydrogenation derivatives as reported by National Institute of Standards and Technology (NIST, https://webbook.nist.gov).

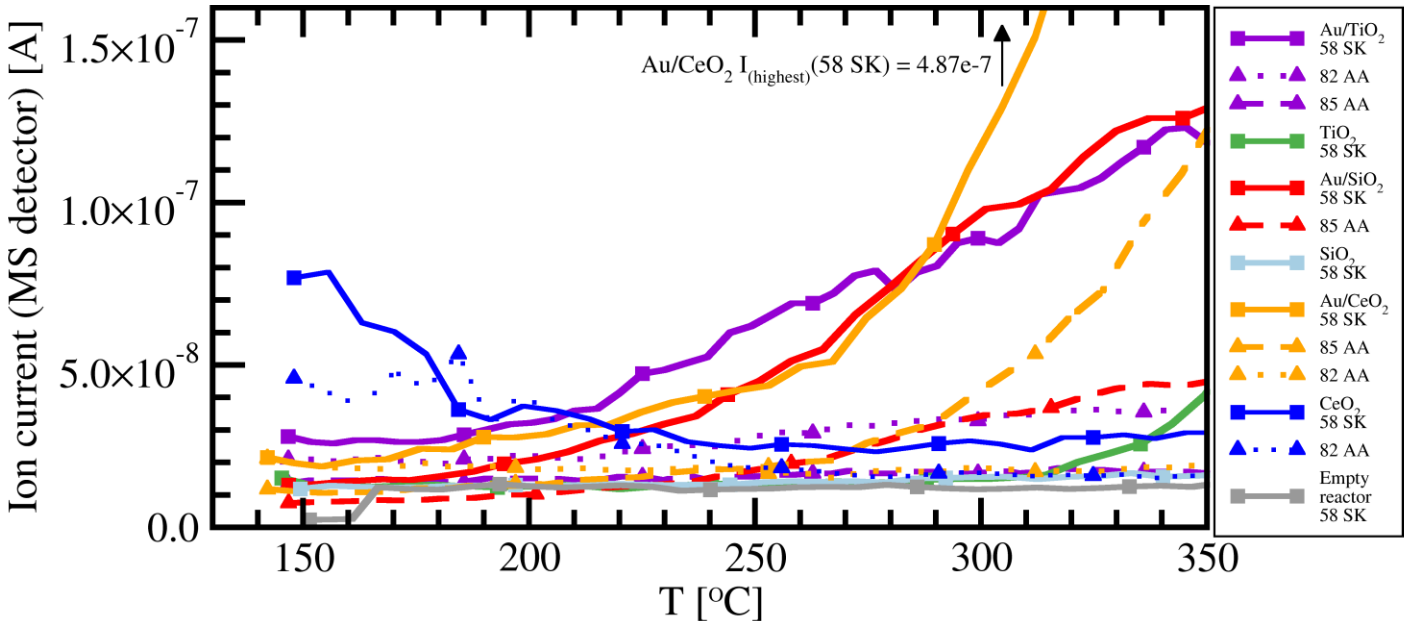


**Figure S 2.** The plot presenting changes of intensity (ion current) of mass spectrometry signals while probing the outlet stream of the reactor fed with vapours of mesityl oxide and a $H_2$/He mixture. The reactor's bed was filled with a layer of a catalyst or a pure oxide support. According to Figure S 1 signal m/z = 58 amu best represents the concentration of sole saturated ketone (SK, i.e. 4-methylpentan-2-one also known as methyl-isobutyl ketone). Signals m/z = 82 and 85 amu best represent the concentration of sole allylic alcohol (4-methylpent-3-en-2-ol).

**Table S 1.** Summary of the composition analysed by gas chromatography of the outlet stream of the reactor containing a layer of a catalyst fed with vapours of mesityl oxide (α-MesOx, 4-methylpent-3-en-2-one, α isomer C=C double bond position) and a $H_2$/He mixture. Meaning of the abbreviations in the columns headers: SK = saturated ketone (4-methylpentan-2-one, methyl-isobutyl ketone); SA = saturated alcohol (4-methylpentan-2-ol); AA = allyl alcohol (4-methylpent-3-en-2-ol); iso = β isomer of MesOx (4-methylpent-4-en-2-one, isopropenyl acetone)

| Sample | % FID signal (peak) area | | | | | Conversion of α-MesOx [%] |
|---|---|---|---|---|---|---|
| | SK | SA | AA | iso | α-MesOx | |
| 8.7%$_{wt.}$ Au/$CeO_2$ | 15.20 | 0.00 | 0.00 | 9.13 | 75.67 | 24.33 |
| 0.5%$_{wt.}$ Au/$TiO_2$ | 3.41 | 0.00 | 0.00 | 12.72 | 83.87 | 16.13 |
| 10.0%$_{wt.}$ Au/$SiO_2$ | 4.86 | 0.00 | 0.00 | 5.01 | 90.14 | 9.86 |
